# Supplementary material for: SARS-CoV-2 Reinfection and Severity of the Disease: A Systematic Review and Meta-Analysis
Source: Viruses. 2023 Apr 14;15(4):967. doi: 10.3390/v15040967 (PMC10145185; doi:10.3390/v15040967)
Supplement: Supplementary file 1 [file viruses-15-00967-s001.zip › viruses-2312497-supplementary.pdf]

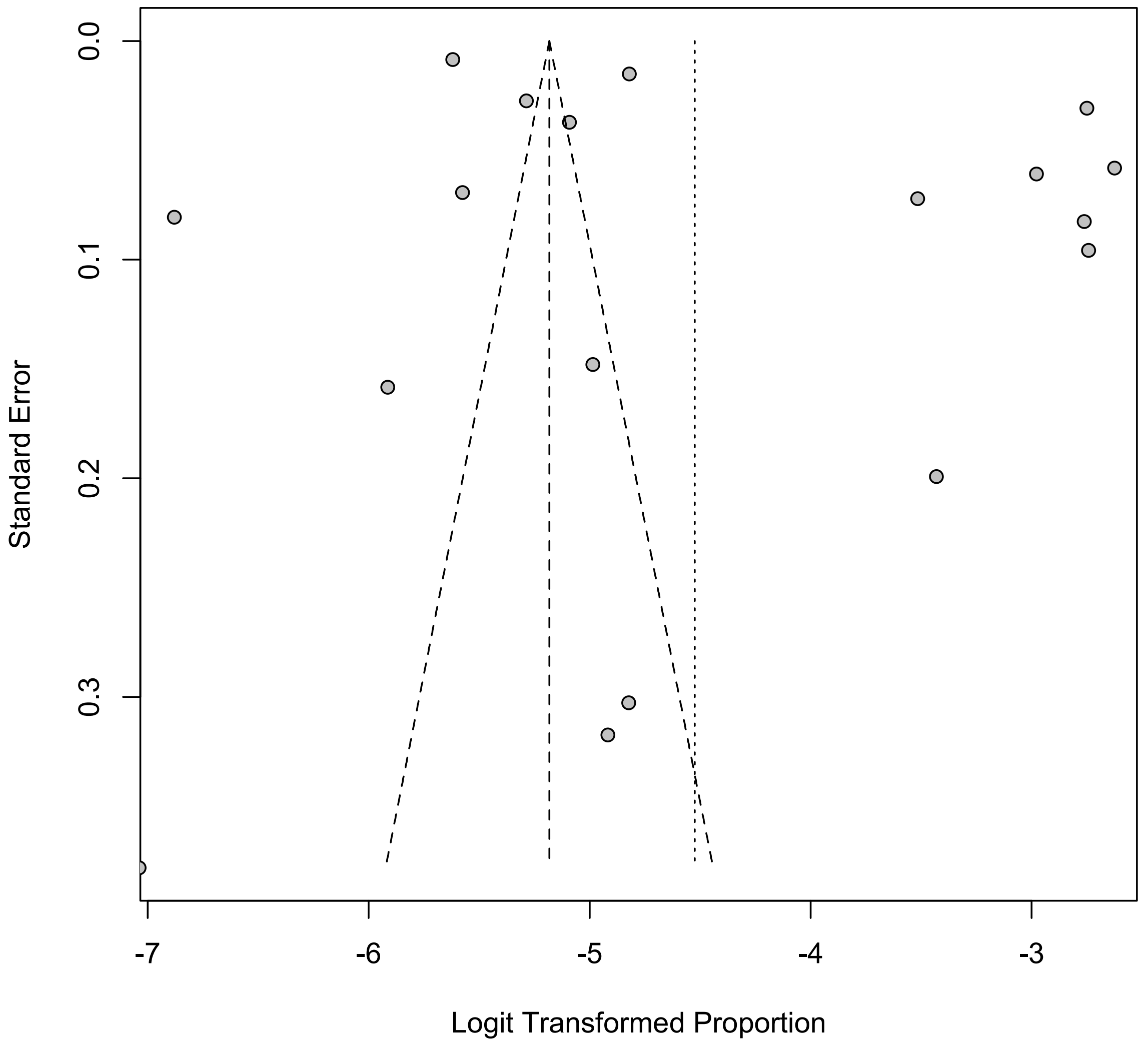

Supplementary Figure S1. Asymmetric funnel plot showing the relationship between the 18 studies included in the metanalysis on SARS-CoV-2 reinfection rate.

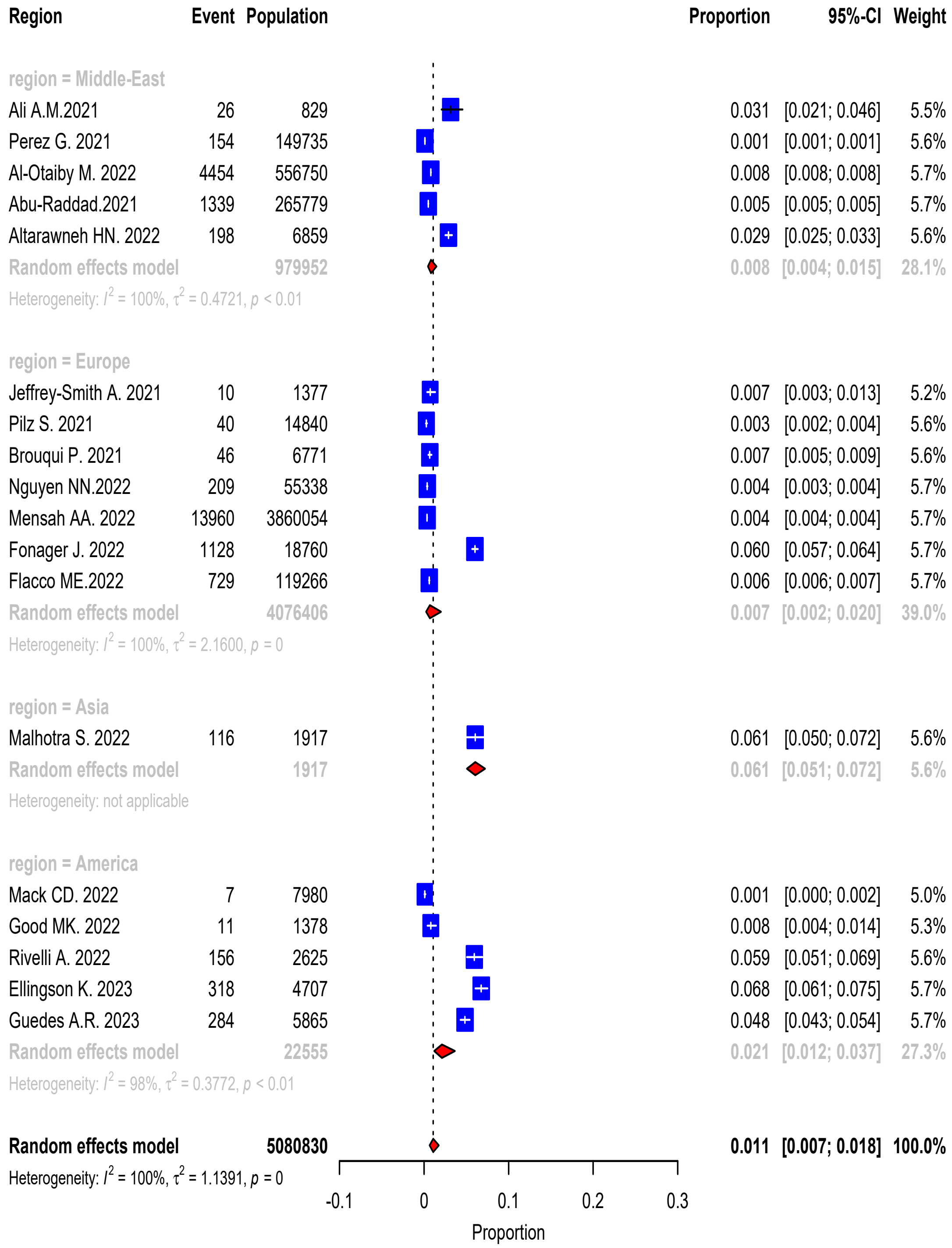

Supplementary Figure S2. Forest plot of SARS-CoV-2 reinfection rate according to region where studies took place (18 cohort studies).

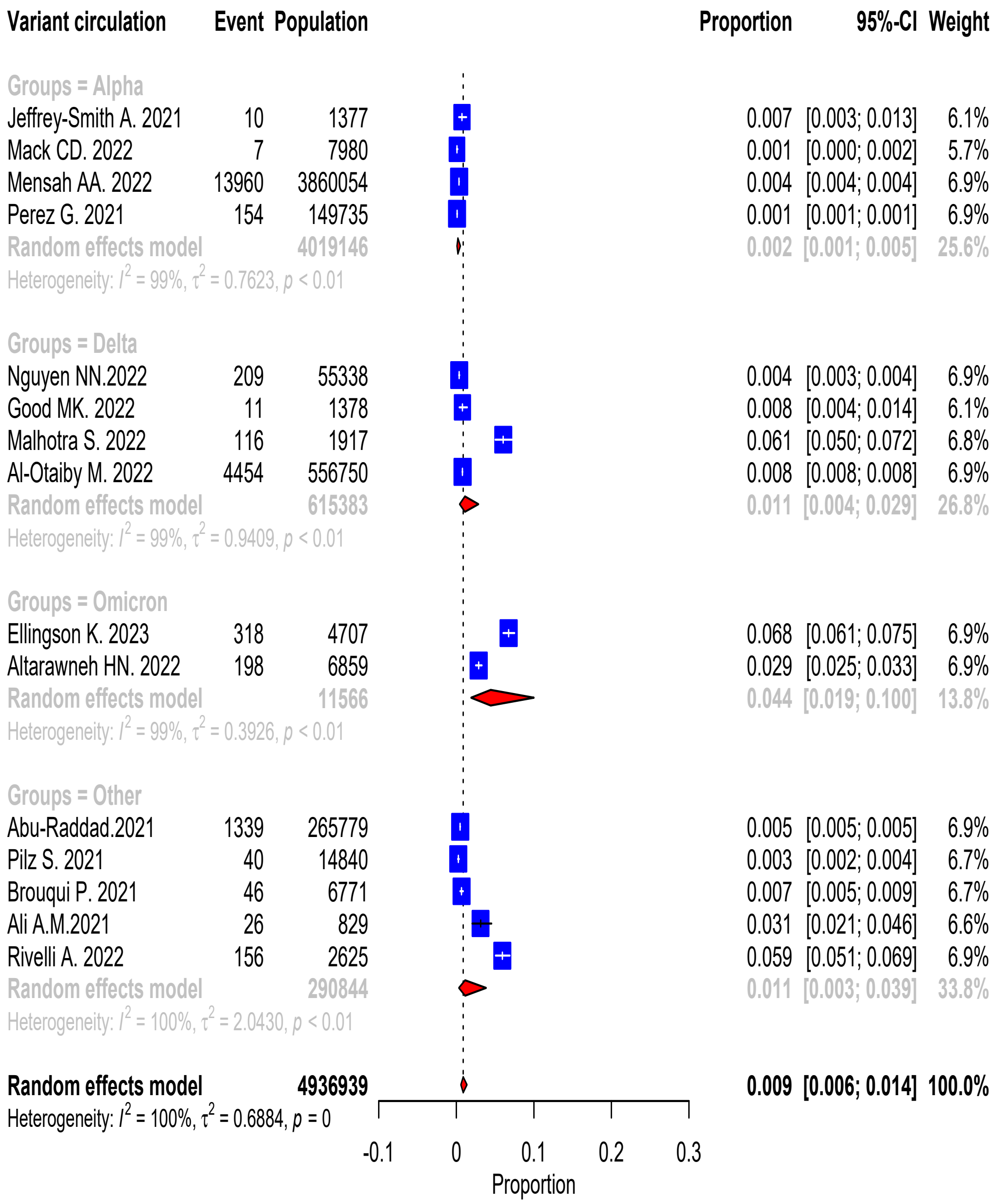

Supplementary Figure S3. Forest plot of SARS-CoV-2 reinfection rate according to most frequent SARS-CoV-2 circulating at the time of reinfection (15 studies).

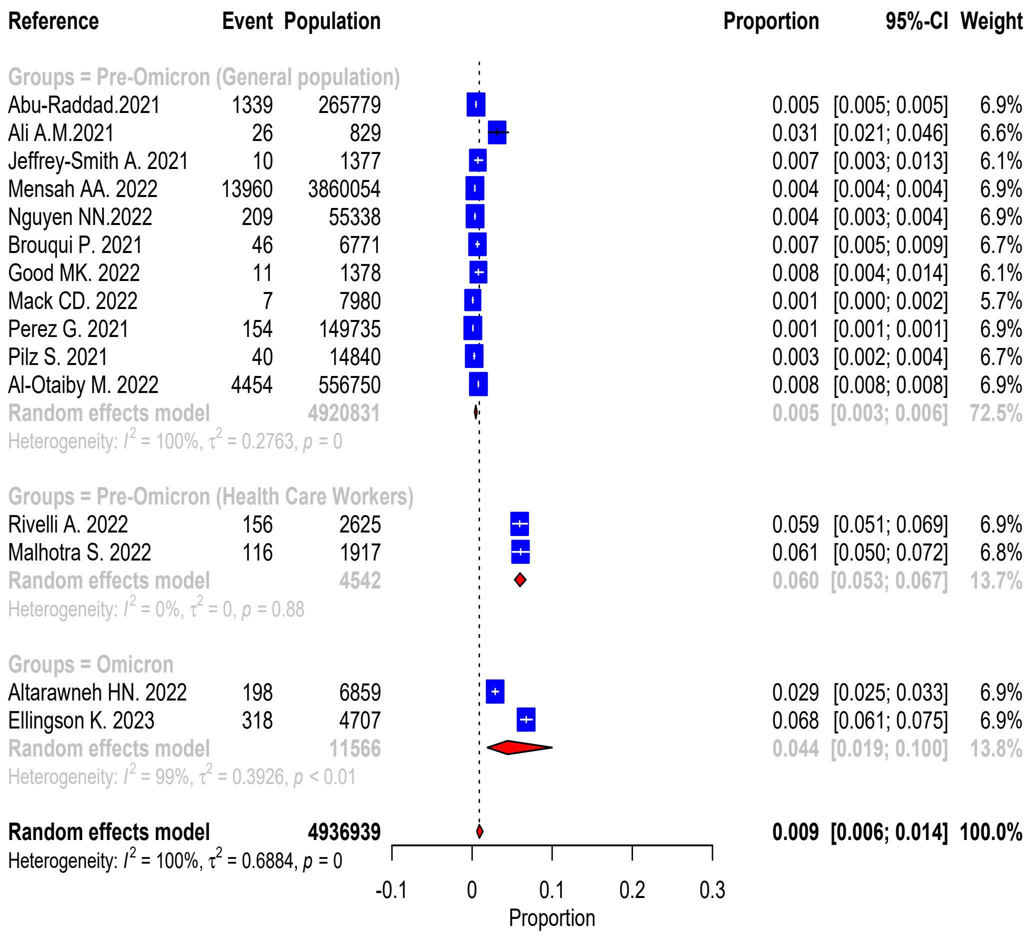

Supplementary Figure S4. Forest plot of SARS-CoV-2 reinfection rate during the pre-Omicron period and Omicron period (15 studies).

Supplementary Table S1. Characteristics of included studies.

Reference	Type of study	Country and setting	Period of study	Number of reinfection cases	Vaccination status in reinfected patients	Number of discharged patients	Vaccination status in discharged patients	Reinfection rate (%)	Discharged patients			Reinfected patients			Length of time between two episodes of infection (days)	Risk factors for reinfection	Most frequent variant circulating at the time of reinfection in the area**	Immunity status before reinfection
									Age (years)	Sex (M/F)	Comorbidities	Age (years)	Sex (M/F)	Comorbidities				
Gupta V. 2021 **[13]**	Case series	India, one hospital, HCWs*	21 August 2020 and 5 September 2020	2	-	-	-	-	-	-	-	25 and 28	1/1	None	Mean ±SD: 109.5±2.1, range: 108-111	Not described	Not available	Not available
Lee J.S. 2020 [14]	Case series	South Korea, three hospitals	18 March 2020 to 16 May 2020	6	-	-	-	-	-	-	-	Mean ±SD: 39.5 ±20.3, range: 17-72	2/4	Allergic rhinitis (n=1, 16.7%) dyslipidaemia (n=1, 16.7%) Parkinson (n=1, 16.7%), depression (n=1, 16.7%)	Mean ±SD: 30.7 ± 8.6, range: 24-47	Not described	Not available	6/6 patients with positive IgG
Salehi-Vaziri. 2021 [15]	Case series	Iran, one hospital	4 July 2020 to 22 August 2020	3	-	-	-	-	-	-	-	Mean ±SD: 42.7 ± 11.0	2/1	Not described	Mean ±SD: 110 ±46.5, range: 63-111	Not described	Not available	2/3 patients with positive IgG
Amoril MR. 2021 [16]	Case series,	Brazil, one hospital, HCW*	10 June 2020 to 5 October 2020	4	-	-	-	-	-	-	-	Mean ±SD: 44.0 ± 11.6	0/4	Chronic bronchitis (n=1, 25.0%)	Mean ±SD: 126 ±50, range: 55-170	Not described	Not available	1/3 patients with positive IgG, 1 with no information

Yu ALF. 2021 [17]	Case series	Brazil, one hospital, HCWs*	17 November 2020 and 1 Februrary 2021	2	1/2 patient vaccinated before the second infection	-	-	-	-	-	-	34 and 41	1/1	Gastroplasty (n=1, 50.0%), chronic respiratory disease (n=1, 50.0%)	Mean ± SD: 159.5 ± 19.1, range: 146-173	Not described	20 J (Gamma, V3)	Not available
Naveca FG. 2021 [18]	Case series	Brazil, one hospital	December 2020 to June 2021	25	2/25 patients vaccinated before the second infection	-	-	-	-	-	-	Age ranged from 17 to 73 years, No information on mean or median	9/16	80% (no detail)	Mean ± SD: 242.5 ± 75.6, range: 92-387	Not described	Delta (21J, 21A 21I)	Not available
Sanyang B. 2021 [19]	Case series	Gambia, one hospital	21 January 2021 and 1 February 2021	2	-	-	-	-	-	-	-	31 and 36	0/2	None	Mean ± SD: 164.5 ± 29.0, range: 144-185	Not described	20 I (Alpha, V1)	Not available
Sonié P. 2022 [20]	Case series	Portugal, one hospital	Before 2021	2	-	-	-	-	-	-	-	60 and 25	1/1	Partial nephrectomy (n=1, 50.0%)	Mean ± SD: 119.5 ± 34.6, range: 95-145	Not described	Delta (21J)	0/2 patients with positive IgG

Abu-Raddad. 2021 [21]	Prospective cohort	Qatar, national study	28 February 2020 to 28 April 2021	1339 reinfections (a PCR-positive swab ≥ 90 days after the first infection), 1304 individuals that could be matched with 6288 primary infection in a 1: 5 ratio by sex, 5 year age group and nationality and PCR test calendar week	-	265 779	All patients were not vaccinated	0.5	Median: 32, range: 26-39 (for 6288 primary infection)	5 499/789 (for 6 288 primary infection)	Not described	Median: 32, range : 26-39 (for 1 304 reinfection cases)	1 130/174 (for 1304 reinfection))	Not described	Median: 277, range:79-315	Not described	20H (Beta, V2)	Not available
Ali A.M. 2021 [22]	Prospective cohort	Iraq, one hospital	May to mid-October 2020	26	-	829	-	3.1	Not described	Not described	Not described	Mean ± SD: 39.6 ± 9.8, range: 14-55 years	14/12	Not described	Not described	Lack of anti-nucleocapside IgG in recovered COVID-19 patients may be associated with reinfection risk	20E (EU1)	1/26 patients with positive IgG
Jeffrey-Smith A. 2021 [23]	Prospective cohort	United Kingdom, 13 care homes	10 April 2020 to 31 January 2021	10 confirmed infections (eight probable, and five possible infection were excluded)	-	1 377 (including 656 previously infected patients and 721 primary infection)	-	0.7	Not described	Not described	Not described	Not described	1/9	Not described	Not described	Not described	20 I (Alpha, V1)	6/10 patients with positive IgG

Mensah AA. 2022 [24]	Prospective cohort	United Kingdom, national study	27 May 2020 to 2 May 2021	13 960	5 858 /13 960 patients vaccinated before the second infection	3 860 054	-	0.4	Median: 39, range: 25-55	1 814 228 / 2045826	Not described	Median: 48 years, range: 31-65	4 607/9 353	Not described	Not described	Increased risk of reinfection was observed in the oldest age categories (70–79 years old and 80+) and in female patients	20 I (Alpha, V1)	Not available
Rivelli A. 2022 [25]	Prospective cohort	United States, a large Midwestern healthcare system (26 hospitals and over 500 healthcare sites in Illinois and Wisconsin), HCWs	1 March 2020 to 10 January 2021	156	-	2 625	-	5.9	Mean ± SD: 38.3±11.6	361/2264	Not described	Mean ± SD: 37.8±10.6	14/142	Not described	Mean ±SD: 141.2 ± 42.8	Not described	21C (Epsilon)	Not available

Nguyen NN. 2022 [26]	Prospective cohort	France, one hospital	19 March 2020 28 August 2021	209	207 patients with available information on vaccination and none was vaccinated	55 338	202 reinfected patients with available information on vaccination, 13/202 vaccinated patient	0.4	Not described	Not described	Not described	Mean ± SD : 40.4 ± 19.8	101/108	177/209 patients with available information on comorbidities, chronic respiratory disease (n=26, 14.7%), chronic heart disease (n=23, 13.0%), hypertension (n=20, 11.3%) obesity (n=20, 11.3%), diabetes (n=12, 6.8%), immunodepression (n=5, 2.8%)	Mean ±SD 232.9 ± 104.0	Not described	Delta (21A)	33/39 patients with positive Ig G
Altarawneh HN. 2022 [27]	Prospective cohort	Qatar, national study	8 June 2022 to 4 July 2022	198, all (reinfected with Omicron BA.4/BA.5)	-	6 859	-	2.9	Not described	Not described	Not described	Median: 36, range: 29-51	88/110	Not described	Not described	Not described	Omicron (22B, 22A, 21L, 22C)	Not available
Brouqui P. 2021 [28]	Prospective cohort	France, one hospital	27 January 2020 to 12 January 2021	46	-	6 771	-	0.7	Not described	Not described	Not described	Mean ± SD: 50.0 ± 22.0	25/21	39/46 patients with information of comorbidities, hypertension (n=8, 20.5%), diabetes (n=5, 12.8%), obesity (n=4, 10.3%), asthma (n=4, 10.3%), chronic heart disease (n=3, 7.7 %)	Mean: 172, range: 90-308	Not described	20A .EU2	Not available
Fonager J. 2022 [29]	Prospective cohort	Denmark, multi centres	29 November 2021 to 2 January 2022	1 128, including 970 BA.1 and 158 BA.2 reinfections	-	18 760, including 16 137 BA.1 and 2 623 BA.2 reinfections	-	6.0	Median 31 for BA.1 and median: 32 for BA.2	Not described	Not described		Not described	Not described	Not described	Not described	Delta (21J, 21I)	Not available

Good MK. 2022 [30]	Prospective cohort	USA, 21 universities	1 January 2021 to 30 November 2021	11	1/11 partially vaccinated and seven/11 fully vaccinated	1 378	92 /1 378 partially vaccinated and 846/1 378 fully vaccinated	0.8	Not described	800/577/1	Not described	Not described	Not described	None	Mean: 197.5, range: 95-301	Not described	Delta (21J, 21I)	Not available
Mack CD. 2022 [31]	Prospective cohort	USA, one hospital	1 March 2020 to 30 May 2021	7	-	7 980	-	0.1	Not described	Not described	Not described	Mean: 30, range: 19-44 years	7/0	Immunocompromised (n=1, 14.3%)	Mean ±SD : 151.7 ± 66.0	Not described	20 I (Alpha, V1)	6/7 patients with positive IgG
Guedes A.R. 2023 [32]	Prospective cohort	Brazil, one hospital	10 March 2020 to 10 March 2022	284, including 281 HCWs	-	5865	281 patients with information on vaccination, including 181 with two doses, 90 with threer doses and 10 unvaccinated	4.8	Not described	Not described	Not described	Median: 39 (range: 30-47 years)	202/82	n=62 (), cardiovascular including hypertension (n= 42, 14.8%), diabetes (n=15, 5.3%), chronic respiratory disease (n=7, 2.5%), malignancy (n=6, 2.1%), obesity (n=5,1.8%), pregnancy (n=1, 0.4%)	Mean 429, range: 122-674 days and 507,(range: 122-674) in the during period	Risk of reinfection was higher during the Omicron period	Omicron (21K, 21L)	Not available
Ellingson K. 2023 [33]	Prospective cohort	USA, 8 Us location in Arizona, HCWs*	December 2021 to April 2022	318, inlcuding only patients reinfected with Omicron variant	5 with one1 dose, 107 with 2 doses and (81) with three doses	4707	34 with one dose, 646 with two doses and 513 with three doses	6.8	Age groups	Not described	489, not described	Age groups	144/174	n= 106, not detailed	Not described	Risk of reinfection was lower in patients with 2 or 3 doses of vaccine	Omicron (21L, 21K, 21C)	Not available
Flacco ME. 2022 [34]	Retrospective cohort	Italy, Abruzzo Region	2 March 2020 to 18 February 2022	729	204 /729 partially vaccinated and 182/729 fully vaccinated before the second infection	119 266	-	0.6	Mean ± SD: 41.6 ± 21.9	58 783/60 483	Hypertension (n=11 069; 9.3%), diabetes (n=5 042; 4.2%), cardiovascular disease (n=9 916, 8.3%), COPD** (n=33 718; 3.1%), kidney disease (n=1 517;1.3%), cancer (n=4 119;3.5%)	Mean ±SD: 35.2 ± 21.6	312/417	Hypertension (n=44, 6.0%), chronic heart disease (n=37, 5.1%), diabetes (n=26, 3.6%), COPD** (n=21, 2.9%), cancer (n=16, 2.2%), chronic kidney disease (n=4, 0.6%)	Mean: 277	Risk of reinfection was higher in female, younger, and unvaccinated patients and during the Omicron wave	Omicron (21K, 21L)	Not available

Perez G. 2021 [35]	Retrospective cohort	Israel, Maccabi Health Care Service computerised database (approximately 25% of the population)	16 March 2020 to 27 January 2021	154	-	149 735	-	0.1	Not described	Not described	Not described	Mean ± SD : 31.5 ±19.7	94/60	Immunocompromised (n=5, 3.2%)	Mean ± SD: 165.7 ± 57.6	Not described	20 I (Alpha, V1)	Not available
Pilz S. 2021 [36]	Retrospective cohort	Austria, national study	1 September 2020 to 30 November 2020	40	-	14 840	-	0.3	Not described	Not described	Not described	Mean ± SD: 44.2 ± 21.5	15/25	Not described	Mean ±SD: 212 ± 25	Not described	20A .EU2	Not available
Malhotra S. 2022 [37]	Retrospective cohort	India, one hospital, HCWs*	3 March 2020 to 18 June 2021	116	39/116 partially vaccinated and 17/116 fully vaccinated before the second infection	1 917	356/1917 partially vaccinated and 1 089/1917 fully vaccinated	6.1	Not described	1 107/810	Not described	Not described	62/54	Not described	Median: 183, range: 13-61	Full vaccination was associated with a lower risk of reinfection	Delta (21J, 21A, 21I)	Not available
Alotaiby M. 2022 [38]	Retrospective cohort	Saudi Arabia, national study	1 March 2020 to 1 December 2021	4454	1474/4454 vaccinated, including 1453 vaccinated before the second infection) and 21 vaccinated the third infection)	556750		0.8	Not described	Not described	Not described	Mean ± SD: 33 ± 12 (second infection) and 33 ± 12 (third infection)	2847/1607	1029/4454, diabetes (n=197, 4.4%), hypertension (n=186, 4.2%), obesity (n=166, 3.7%), respiratory disease (n=132, 3.0%), cardiovascular disease (n=118, 2.6%), cancer (n=54,1.2%), HIV (n=43, 1.0%), pregnancy (n=43), , chronic kidney disease (n=26, n=0.6%), immunosuppressive drug (n=25, n=0.6%), sickle cell anaemi a (n=23, 0.5%), history of stroke (n=8, 0.2%), organ transplant (n=8, 0.2%)	Not described	Risk of reinfection was higher in patients with commorbidities and in those reinfected with delta variant. Risk of reinfection was lower in vaccinated patients	Delta (21J)	Not available

(*) HCW = healthcare workers, (**) COPD = chronic obstructive pulmonary disease

(**) Most frequent variant circulating at the time of reinfection in the area (information available from: https://covariants.org/)

Supplementary Table S2. NICE quality assessment for case series.

Study	Was the study question or objective clearly stated?	Was the study population clearly and fully described, including a case definition?	Were the cases consecutive?	Were the patients comparable?	Was the intervention clearly described?	Were the outcomes measured clearly defined, valid, reliable, and implemented consistently across all study participants?	Was the length of follow-up adequate?	Were the statistical methods well-described?	Were the results well-described?	Total score	Total quality score
Gupta V. 2021	1	1	1	NA	1	1	1	NA	1	7	Good
Lee J.S. 2020	1	1	1	NA	1	1	1	NA	1	7	Good
Salehi-Vaziri.2021	1	1	1	NA	1	1	1	NA	1	7	Good
Yu ALF.2021	1	1	1	NA	1	1	1	NA	1	7	Good
Amoril MR.2021	1	1	1	NA	1	1	1	NA	1	7	Good
Naveca FG. 2021	1	1	1	NA	1	1	1	1	1	8	Good
Sanyang B. 2021	1	1	1	NA	1	1	1	NA	1	7	Good
Sonié P. 2022	1	1	1	NA	1	1	1	NA	1	7	Good

Supplementary Table S3. Newcastle-Ottawa Scale (NOS) quality assessment of cohort studies.

Studies	Selection				Comparability	Exposure			Total score	Total quality score
Author, year	Representativeness of the exposed cohort	Selection of the non-exposes cohort	Ascertainment of exposure	Outcome of Interest was not present at start of study	Comparability	Assessment of outcome	Sufficient follow-up	Adequacy of follow-up of cohort		
Abu-Raddad.2021	1	1	1	1	1	1	1	1	8	Good
Ali A.M.2021	1	0	1	1	NA	1	1	1	6	Fair
Jeffrey-Smith A. 2021	1	0	1	1	NA	1	NA	NA	4	Fair
Mensah AA. 2022	1	1	1	1	1	1	1	1	8	Good
Rivelli A. 2022	1	0	1	1	NA	1	1	1	6	Fair
Nguyen NN.2022	1	1	1	1	1	1	1	1	8	Good
Altarawneh HN. 2022	1	1	1	1	1	1	NA	1	7	Good
Brouqui P. 2021	1	0	1	1	NA	1	1	1	6	Fair
Fonager J. 2022	1	0	1	1	1	1	NA	1	6	Fair
Good MK.	1	0	1	1	NA	1	1	1	6	Fair

2022										
Mack CD. 2022	1	0	1	1	NA	1	NA	NA	4	Fair
Guedes A.R. 2023	1	0	1	1	NA	1	1	1	6	Fair
Ellingson K. 2023	1	1	1	1	NA	1	1	1	7	Good
Flacco ME.2022	1	1	1	1	NA	1	1	1	7	Good
Perez G. 2021	1	0	1	1	NA	1	1	1	6	Fair
Pilz S. 2021	1	0	1	1	NA	1	1	1	6	Fair
Malhotra S. 2022	1	0	1	1	1	1	NA	1	6	Fair
Al-Otaiby M. 2022	1	1	1	1	NA	1	1	1	7	Good

Supplementary Table S4. SARS-CoV-2 variant among 2354 reinfected patients.

Reference	Patient number with identified variant	Variant for the second infection
Yu ALF. 2021 [17]	2	B.1.1.28 and one patient with mutation E484K
Naveca FG. 2021 [18]	25	VOc Gama (lignage P.1, n=21) Gamma plus with linage P.1.1.7 (n=2) P.1.1.2 (n=1) and P.1.14 (n=1)
Sanyang B. 2021 [19]	2	Lineage B.1.235 and linage B.1
Abu-Raddad. 2021 [21]	683	B.1.351 (Beta, n=413), B.1.1.7 (Alpha, n=57), wilde-type (n=213)
Jeffrey-Smith A. 2021 [23]	7	7/10 patients, 6 patients reinfected with alpha (B.1.1.7) and one patient with B.1.36.28
Nguyen NN. 2022 [26]	159	21A Delta (B.1.617.2, n=100), 20A.EU2 (B.1.160, n=34), 20I (Alpha.V1, B.1.1.7, n=25)
Altarawneh HN. 2022 [27]	198	Omicron BA.4 and/ or BA.5
Mack CD. 2022 [31]	3	B.1.2 (n=1), B.1.1.7, (n=1), B.0.1.2 (n=1)
Ellingson K. 2023 [33]	318	Omicron variant
Alotaiby M. 2022 [38]	957	Delta (844), beta (49), alpha (29), wild-type (35)

Supplementary Table S5. Prevalence of comorbidities in 5873 reinfected patients.

Symptoms	Number	Proportion
Hypertension	258	4.4
Diabetes	255	4.3
Obesity	195	3.3
Chronic respiratory disease	192	3.3
Heart disease	181	3.1
COPD	48	0.8
Pregnancy	44	0.7
HIV	43	0.7
Heart disease including hypertension	42	0.7
Immunocompromised	36	0.6
Kidney disease	30	0.5
Organ transplant	8	0.1
History of stroke	8	0.1
Asthma	4	0.1
Partial nephrectomy	1	0.0
Parkinson	1	0.0
Gastroplasty	1	0.0
Dyslipidemia	1	0.0
Depression	1	0.0
chronic bronchitis	1	0.0
Allergic rhinitis	1	0.0
